# Reconstructive considerations in head and neck surgical oncology: United Kingdom National Multidisciplinary Guidelines

**DOI:** 10.1017/S0022215116000621

**Published:** 2016-05

**Authors:** M Ragbir, J S Brown, H Mehanna

**Affiliations:** 1 Newcastle upon Tyne Hospitals NHS Foundation Trust, Newcastle upon Tyne, UK; 2 Department of Oral and Maxillofacial Surgery, Aintree University Hospitals NHS Foundation Trust, Liverpool University, Liverpool, UK; 3 School of Cancer Sciences, Institute of Head and Neck Studies and Education, University of Birmingham, University Hospital Birmingham, Heart of England NHS Foundation Trust, Birmingham, UK

## Abstract

This is the official guideline endorsed by the specialty associations involved in the care of head and neck cancer patients in the UK. The reconstructive needs following ablative surgery for head and neck cancer are unique and require close attention to both form and function. The vast experience accrued with microvascular reconstructive surgery has meant a significant expansion in the options available. This paper discusses the options for reconstruction available following ablative surgery for head and neck cancer and offers recommendations for reconstruction in the various settings.

**Recommendations:**

• Microsurgical free flap reconstruction should be the primary reconstructive option for most defects of the head and neck that need tissue transfer. (R)

• Free flaps should be offered as first choice of reconstruction for all patients needing circumferential pharyngoesophageal reconstruction. (R)

• Free flap reconstruction should be offered for patients with class III or higher defects of the maxilla. (R)

• Composite free tissue transfer should be offered as first choice to all patients needing mandibular reconstruction. (R)

• Patients undergoing salvage total laryngectomy should be offered vascularised flap reconstruction to reduce pharyngocutaneous fistula rates. (R)

## Introduction

The problems of reconstructive surgery for the head and neck are variable and can be very complex.^1^^,^^2^ These guidelines have been divided into the management of the loss of skin, the maxilla, the mandible, including the associated soft tissues, the oropharynx and the laryngopharynx. There is very little level 1 evidence relating to the reconstruction of head and neck defects. Mandibular reconstruction techniques are fairly standard but some controversy remains regarding the midface and maxilla because of the complexity of the defects and the possibility of using a dental or facial prosthesis.

Most reconstructions are performed primarily following tumour extirpation, but secondary reconstructions are also undertaken to treat problems such as fistulae or osteoradionecrosis. Modern techniques aim for one stage reconstruction utilising vascularised tissues with a high success rate and good overall results.

Priorities of reconstruction include restoring oral cavity lining, maintaining oral competence, maintaining function of speech and swallowing and providing an acceptable aesthetic result. Choice of reconstructive options depends on patient comorbidities, factors relating to the surgical defect, any future possible treatments including radiotherapy and donor site morbidity. No appropriately powered randomised controlled trials exist to determine flap selection in most instances and this is usually determined by the expertise of the individual surgeon. Patient factors include prior treatments, especially surgery and radiotherapy and the patient's overall health including medical and social history. Multiple tissue types often require to be reconstructed.

## Oral cavity soft tissues

Oral soft tissues include tongue, floor of mouth, buccal mucosa and the retro-molar trigone extending to the tonsillar area. It is rare that only one of these areas is involved. Reconstructive access is usually determined by the extent of surgical resection and may involve a lip-split and mandibular osteotomy, although a per-oral approach is usually possible.

Microsurgical techniques provide the mainstay of oral soft tissue reconstructions as they allow importation of large volumes of healthy tissue from sites distant to prior surgical or radiotherapy fields. Flaps commonly used include the radial forearm flap (RFF) and the anterolateral thigh (ALT) flap. Less commonly the latissimus dorsi, rectus abdominus and flaps based on the scapular and/or para-scapular axis are utilised. More recently, the medial sural artery perforator flap (MSAP) and the superficial circumflex iliac artery perforator flap are being used. The first two represent the workhorse flaps in this field and will be discussed separately.

The RFF allows for importation of a large, thin, pliable flap with excellent reliability and simplicity of harvest.^3^ Multiple skin paddles can be designed and the flap can be raised as a cutaneous, fasciocutaneous, fascial, adipofascial, osseo-fascial or osseo-cutaneous flap (see below). The principal disadvantage of this flap is the poor donor site aesthetics when skin grafting is required.

The ALT flap allows for importation of very large tissue volumes and is versatile.^4^ Fascio-cutaneous and fascial flaps can be raised, along with muscle and fascia lata if required. The flap has a long pedicle, but can be technically challenging to raise. It is a relatively thick flap which can be thinned. If multiple perforating vessels are available, then the flap can be raised with two skin paddles. Donor site morbidity is minimal and use of the ALT is increasing in most reconstructive centres.

If microsurgery is considered, inadvisable local or regional flaps are still used. Within the oral cavity local mucosal flaps can be useful to help close small defects. Regional flaps such as pectoralis major and deltopectoral can be effective in importing tissue, but are not generally considered as a first choice.

## Mandible

Reconstruction of the mandible must address the site and size of the bony defect, associated soft tissue loss and the desirability of dental rehabilitation. Free tissue transfer is the mainstay of mandibular reconstruction as it allows importation of bone which can be tailored to fit the desired shape, is well vascularised and is amenable to osseo-integration. Several flaps are commonly used with high success rates, including the fibula flap, deep circumflex iliac artery (DCIA) flap, scapular flap and RFF.^5^

The fibular flap allows harvest of a long piece of bone which is of adequate height for osseo-integration and can be osteotomised several times for contouring.^6^^,^^7^ This is now made easier with the availability of software to plan the osteotomies at the mandible and on the fibula prior to transfer. It is relatively easy to harvest as an osseus or osteoseptocutaneous flap, with or without muscle. This versatility means it is the workhorse for mandibular reconstruction in most centres. One drawback of the flap is its relative lack of height.

The DCIA flap provides for a high bony segment and the natural curve of the ilium lends itself to lateral mandibular defects where an osteotomy may not be necessary. The donor site defect can be problematic and its skin paddle is usually reserved for external use although muscle can be incorporated for oral reconstruction.

The scapular flap allows for harvest of a relatively small amount of bone. The main advantage of this flap is the large volume of skin and muscle (latissimus dorsi) which can be used. The bone is a good height, but two-team flap harvesting is generally not possible.

Radial forearm flap is rarely used for bone reconstruction as only a small volume of bone of low height can be harvested. There is a risk of subsequent fracture of the radius.

**Fig. 1:**
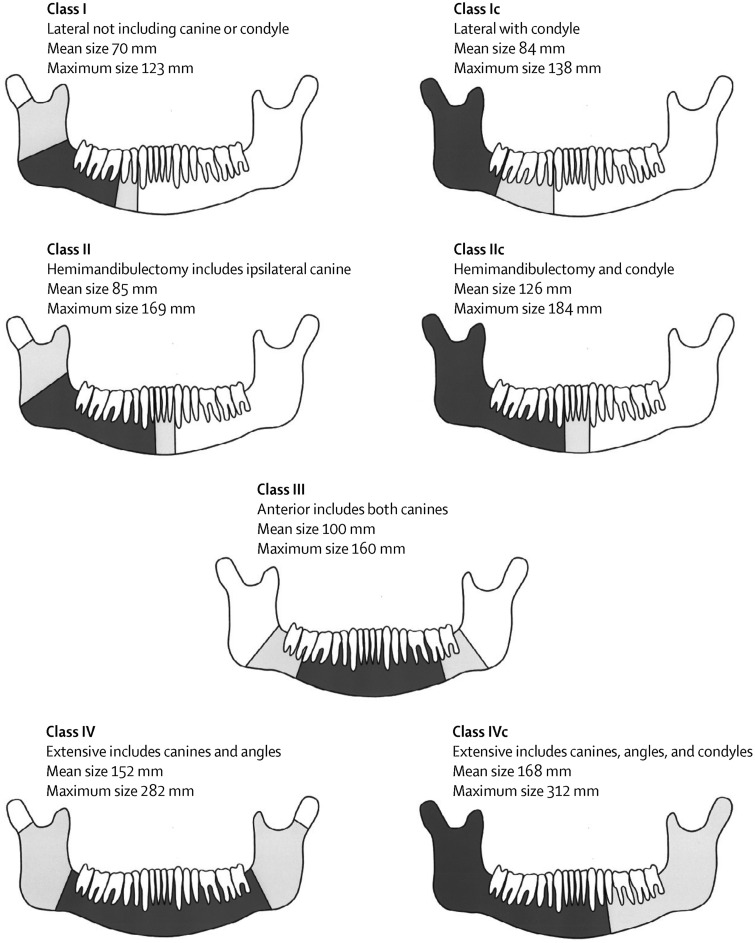
Classification of mandibular defects.

A new classification of the mandibular defect has been described based on the four corners of the mandible which are both angles and both canines (Figure 1):^8^

• Class I (70 mm)/Ic (84 mm): Subcondylar region to the ipsilateral canine and class Ic includes the condyle. Most of the flaps described above will work well as the length of this defect is around 7–8 cms and so all bone donor sites are adequate. In the lateral defect the height of the reconstruction is less problematic.
• Class II (85 mm)/IIc (126 mm): Hemimandibulectomy from subcondylar region including ipsilateral canine and class IIc includes condyle. The iliac crest can work well as the shape of the ipsilateral hip may reduce osteotomy preparation and a scapula may not be sufficiently long for a class IIc when soft tissue is seldom an issue.
• Class III (100 mm): Includes both canines, but neither angle. The choice of flap depends more on the plan of rehabilitation and height of chin support. The fibula flap can be double-barrelled to increase height, but scapula and radius are often difficult to implant successfully for complete oral rehabilitation.
• Class IV (152 mm)/IVc (168 mm): This is an extensive mandibulectomy including at least one angle and both canines. The fibula flap is usually the best option for faithful reconstruction, but the mandible is often best made smaller for such major resections especially if there is loss of maxillary teeth.

Dental rehabilitation is a key part of mandibular reconstruction and pre-operative liaison with an appropriate team including consideration of osseo-integrated implants is mandatory.

## Maxilla and midface

The level of evidence is very weak in all areas of reconstruction, but more particularly in the maxilla and midface because of the differing complexity of the defects, and the potential for skull base involvement.

**Fig. 2:**
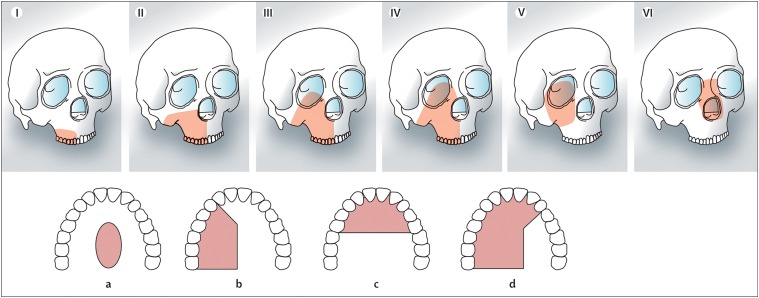
Classification of the maxillary and midface defects. Classes I–VI relate to the vertical component of the defect including orbitomaxillary (class V) and nasomaxillary (class VI) when often the palate and dental alveolus are intact. Classes a–d relate to the increasing size of the palatal and dento-alveolar part of the defect indicating increasing difficulty in obtaining good results with obturation.

Throughout this section, it is necessary to refer to the classification suggested in Fig. 2.^9^ The choice of a prosthetic option or reconstruction depends on the nature of the defect. In class I and II defects an obturator is a reasonable option, but this becomes less favourable as the orbital adnexae are involved (class III), orbital exenteration (class IV) and the midface defects of an orbitomaxillary (class V) or nasomaxillary (class VI) nature. This refers not only to the vertical component but also to the extent of the dental or alveolar part of the resection relevant to the prosthodontist in deciding on appropriate obturation. Other classifications suggested include those by Okay *et al*., but there is no distinction between classes III and IV.

All cases involving the loss or ablation of the maxilla and/or midface should be discussed in a multidisciplinary setting. The choice of reconstruction or prosthetics requires discussion among the ablative and reconstructive teams, the prosthodontist, maxillofacial technician, the patient and the family. There are clear advantages in simplifying the surgery and using prosthetic options, but this choice becomes more difficult to deliver and for the patient to cope as the defect becomes larger and more complex.

*Class I*: This includes resections of the alveolar bone not resulting in an oroantral fistula and these can either be left to granulate or treated with a local flap. Also included are defects involving the junction of the hard and soft palate usually obturated or reconstructed with a soft tissue flap, and minor maxillectomies which may occur following the removal of small inverted papillomas which generally do not require rehabilitation.

*Class II*: This is the standard hemimaxillectomy not involving the orbital floor or adnexae. Obturation is often very successful for this form of defect as the orbit does not require support and if the defect is not too large there is less of a problem for the patient in terms of retention and stability of the prosthesis. In more extensive cases (classes IIc–d), it is possible to gain very good retention with an implant-retained prosthesis, although reconstruction with the fibula flap has also shown good outcomes. A vascularised bone with greater height, such as the DCIA flap which includes the iliac crest and internal oblique muscle, will give better support to the peri-nasal area. The scapula flap can be supplied by the circumflex scapular artery which supplies the lateral scapula (scapula flap) through peri-osteal perforators along its length or the angular branch of the thoracodorsal artery which supplies the scapula tip. The advantage of the scapula tip option is that the pedicle is considerably longer than the circumflex scapula artery option which is a great advantage in the maxilla and midface as the recipient vessels are more distant.

*Class III*: In these cases, there is loss of the orbital support and often a part of the nasal bones may also require reconstruction. There is good consensus in the literature that the restoration of orbital support with vascularised tissue (pedicled or free flap) is essential to ensure healing of the bone graft and reduce the soft tissue problems such as epiphora and ectropion. The iliac crest with internal oblique provides the best solution if an implant-retained prosthesis is planned, but the scapula tip flap using latissimus dorsi muscle is also a good option with a more reliable pedicle. The fibula is also described for this defect but considerable skill in the adaptation of this flap for the defect is required with variable results. The rectus abdominus with non-vascularised bone is also an option but is associated with a high ectropion rate and there is a risk of bone loss if radiotherapy is required. The vastus lateralis based on the descending branch of the lateral circumflex femoral artery is another option.

Obturation alone will result in facial collapse, poor support of the orbit and a high risk of vertical orbital dystopia and ectropion. In children, the scapula tip will probably be the best option as the iliac crest has a cartilaginous cover and the vessels are much smaller.

*Class IV*: Reasonable results can be achieved with a soft tissue flap alone such as rectus abdominus or vastus lateralis but this will result in poor definition of the orbital defect and some facial collapse. The choice is similar to class III in that the iliac crest with internal oblique offers better implant options but the scapula tip flap is also a good option.

*Class V*: In the orbitomaxillary defect, the main aim is not to obturate the orbital space with too much soft tissue so as to allow space for an orbital prosthesis. The temporalis or temporoparietal flap are ideal, but in more extensive defects it is worth considering the radial or ALT in a thinner patient.

*Class VI*: If there is loss of the facial skin between the orbits and nasal bones, then free tissue transfer is probably essential. The composite RFF can be ideal if harvested with fascia to line the nasal side of the radial strut and the skin to restore the face. The composite radial can be augmented with a glabella or forehead flap. A classical rhinectomy can be rehabilitated with a prosthesis and of course the surgeon can check the margins of resection and resect more tissue if required. There are very successful full rhinectomy reconstructions performed which can give a permanent biological solution if preferred. In this defect attention must be paid to the restoration of the nasal bones with vascularised tissue to prevent complications during and following radiotherapy.

## Oropharyngeal reconstruction

The oropharynx can be divided into the walls of the oropharynx (lateral and posterior), the base of the tongue and the soft palate. The oropharynx is a muscular tube connecting the larynx and hypopharynx to the oral cavity. The role of reconstruction is to try and maintain the function of the residual tissue. From a functional point of view the most difficult area is the posterior tongue which allows normal movement of the epiglottis and maintains swallowing and speech. The use of transoral robotic and laser resections without reconstruction may give better functional results than reconstructing this muscular tube with non-sensate skin such as the radial forearm flap.

### Reconstruction of the soft palate

The most commonly described method of soft palate reconstruction involves the use of the RFF often in combination with a local flap such as the superiorly based pharyngeal flap or the superior constrictor advancement flap. Some suggest the use of a folded RFF which is de-epithelialised in order to be sutured to the de-epithelialised posterior pharyngeal wall, but a superiorly based pharyngeal flap can be utllised to provide the nasal lining with good results.^10^^,^^11^ The free flap is used in the horizontal part of the defect only if it is possible to close the posterior tongue to narrow the pharynx and maintain its function.

### Reconstruction of the pharyngeal walls and tonsillar regions

Placing free tissue transfers will disrupt the muscular tube and probably decrease function. For this reason, transoral robotic and laser resections are preferred to address these tumours where possible.

### Reconstruction of the posterior tongue

Most surgeons do not claim to be able to restore function in this region if more than half of the posterior tongue requires resection (Table I).

**Table I:** Methods of soft palate reconstruction

No reconstruction	Obturation
Local flaps	Superiorly based pharyngeal
	Palatoplasty and lateral pharyngeal
	Palatal island mucoperiosteal
	Palatal island and pharyngeal
	Masseter and buccal mucosa transposition
	Masseter, buccal mucosa and pharyngeal
	Temporalis
	Superior constrictor advancement
	Velopharyngoplasty or masseter and buccal advancement
Pedicled flaps	Temporal osteocutaneous island
	Galeo-peri-cranial
Free flaps	Radial forearm
	Radial forearm and additional local
	Folded radial forearm
	Lateral arm
	Jejunum
	Anterolateral thigh

## Pharyngo-laryngectomy reconstruction

### Partial pharyngeal defects

Partial pharyngeal defects with more than 3.5 cm of remaining pharyngeal mucosal width may be closed primarily. Defects with less than 3.5 cm of pharyngeal mucosal width remaining may be reconstructed using a pedicled flap – usually a pectoralis major myocutaneous flap. Free flaps, such as radial forearm free flaps, may also be used. If the pharyngeal mucosal remnant is very narrow (<1 cm in width), then it is often better to excise the remnant and undertake a total circumferential reconstruction.

### Total circumferential pharyngolaryngectomy defects

#### Lower anastamosis above clavicles

Where the lower anastamosis of a total circumferential pharyngolaryngectomy reconstruction would lie above the clavicle, several options exist:^12^ jejunal free flap (JFF), gastro-omental free flap (GFF), tubed radial forearm free flap (RFFF) and tubed anterolateral thigh free flap (ALTF). All of the above options carry the risk of free flap failure, anastamotic leaks, anastamotic strictures, donor site morbidity, failure of voice rehabilitation, swallowing problems and a small peri-operative mortality rate.

#### Previously untreated cases

In previously untreated cases, ALTs, tubed over a salivary bypass tube, appear to provide the lowest complication rates – with minimal donor site morbidity, lower leak rates and lower stenosis rates. Good swallowing and voice rehabilitation have also been reported. Alternatives include the JFF^13^ and the RFF. Swallowing problems due to hyper-peristalsis and a ‘wet’ sounding voice are common with JFF, which also carries a morbidity rate due to abdominal complications (≈5 per cent). Radial forearm flap carries lower donor morbidity rates, but higher stenosis and leak rates than JFF. Tubing of the RFF over a salivary bypass tube appears to decrease fistula rates.^14^

#### Post-chemoradiotherapy (salvage) cases

In general, reconstructive free flap surgery in the salvage setting carries higher risks of complications due to the deleterious effects of chemoradiotherapy on tissue vascularity and wound healing. In such cases, limited case series suggest that use of GFFs may have an advantage due to the availability of the omentum. This can be wrapped around the anastamotic site to decrease the possibility of leakage and also improve the overlying skin quality. Additional vascularised tissue can be included with the ALT as a chimaeric flap to resurface the neck in cases where there is poor quality skin or contracted skin that would not safely close post-operatively.

Any of the other options mentioned previously, for example JFF, RFF, may also be used in salvage surgery.

#### Lower anastamosis below clavicles

If the resection extends to below the level of the clavicles, then a gastric pull through or colonic transposition flap may be used. Both these techniques carry significant morbidity and mortality due to the need to enter three visceral cavities. Gastric pull through carries a mortality rate of 5–15 per cent, morbidity of 30–55 per cent and reported fistula rates of 3–23 per cent. Colonic transposition carries similar risks, and appears to be less commonly used. It can however provide a higher reach than gastric pull through, and is therefore useful for tumours that extend up high into the oropharynx.

## Vascularised tissue after salvage laryngectomy

Pharyngocutaneous fistulae (PCF) are known to occur in nearly one-third of patients who undergo salvage total laryngectomy after chemoradiation. Pharyngocutaneous fistulae have severe impact on duration of admission and costs, quality of life and can even cause severe complications such as bleeding, infection and death. Recent meta-analyses suggest that there is a clear advantage in using vascularised tissue from outside the radiation field in the laryngectomy defect, either as a buttress or to augment the circumference of the pharynx.^15^^,^^16^ This intervention reduces the risk of PCF by one-third to a half.

**Recommendations**

• Microsurgical free flap reconstruction should be the primary reconstructive option for most defects of the head and neck that need tissue transfer (R)
• Free flaps should be offered as first choice of reconstruction for all patients needing circumferential pharyngoesophageal reconstruction (R)
• Free flap reconstruction should be offered for patients with class III or higher defects of the maxilla (R)
• Composite free tissue transfer should be offered as first choice to all patients needing mandibular reconstruction (R)
• Patients undergoing salvage total laryngectomy should be offered vascularised flap reconstruction to reduce pharyngocutaneous fistula rates (R)

### Key points

*Mandible and oral cavity*

• The radial forearm and the anterolateral thigh free flaps are the preferred options for oral soft tissue reconstruction. Newer flaps such as the medial sural artery perforator flaps are increasing in popularity
• The fibula free flap is now considered the workhorse for mandibular reconstruction following ablative surgery. Planning software makes osteotomies easier
• The deep circumflex iliac artery with internal oblique provides a superior form for the mandible and facilitates deeper implant placement and should be considered if implant-retained oral rehabilitation is planned
• The scapula provides a good option for extensive soft tissue resections including the mandible and an alternative if atheroma precludes use of the fibula. The donor site is also the best tolerated

*Midface and maxilla*

• Multidisciplinary decision-making should include the patient, surgeon and dental prosthodontist
• Prosthetic options reduce the morbidity of treatment and can give excellent results but reconstructive options should be considered as the defect becomes larger and more complex

*Oropharynx*

• Using local tissue only to restore the constrictor tube is essential. Free tissue transfer is best reserved for the reconstruction of the soft palate
• Functional results for posterior tongue reconstruction are disappointing
• The greater role played by transoral surgery will reduce the need for reconstruction in this area

*Pharyngolarynx*

• Partial pharyngeal defects may be closed primarily or using a pedicled myocutaneous, usually a pectoralis major flap or with a free flap
• Total circumferential defects where the lower anastamosis is above the clavicle can be reconstructed with several free flaps. In previously untreated patients, anterolateral thigh free flaps, tubed over a salivary bypass tube, appear to carry lowest complication rates. In post-radiotherapy patients, limited evidence suggests that gastromental free flaps may have some advantages
• Tubing over and use of a salivary bypass tube appears to decrease complication rates with anterolateral thigh and radial forearm free flaps
• Total circumferential defects where the lower anastamosis is below the clavicle may be reconstructed by gastric pull through or colonic transposition

*Salvage laryngectomy*

• Use of vascularised tissue to buttress or augment the pharynx in patients undergoing salvage total laryngectomy reduces pharyngocutaneous fistula rates
